# SGLT2 and cancer

**DOI:** 10.1007/s00424-020-02448-4

**Published:** 2020-08-20

**Authors:** Ernest M. Wright

**Affiliations:** Physiology Department, David Geffen School of Medicine at UCLA, Los Angeles, CA 90095-1751 USA

**Keywords:** SGLT2, Glioblastoma, PET, Inhibitors

## Abstract

Glycolysis plays a central role in tumor metabolism and growth, and this is reflected in a high rate of glucose uptake. It is commonly assumed that the upregulation of the facilitated glucose transporter GLUT1 meets the tumor’s demand for sugar. This underlies the success in using 2FDG PET imaging in the clinic to identify and stage many tumors. However, 2FDG is not a substrate for a second class of glucose transporters, the sodium-dependent glucose cotransporters, SGLTs, and so 2FDG PET may not provide a complete picture. A specific new radiotracer to detect SGLT activity has been introduced, Me4FDG, and this provides an opportunity to explore the potential role of SGLTs in supporting tumor glycolysis. In this brief review, I highlight the development of Me4FDG and our preliminary studies of Me4FDG PET in cancer patients. We find that the renal isoform, SGLT2, is expressed in pancreatic and prostate tumors and glioblastomas, and Me4FDG PET introduces a new method to image tumors. As SGLT2 drugs are successful in treating type 2 diabetes mellitus, they may also provide a new therapy.

## Introduction

Tumors require glucose to support their high rate of glycolysis to fuel their metabolism, growth, and proliferation. In vitro experiments have shown that withdrawing glucose or inhibiting glycolysis reduces cell proliferation and tumorigenesis. The increased cellular demand for glucose has been linked to the overexpression of the glucose transporter GLUT1 in the SLC2A gene family [4]. This has been exploited to image and stage tumors using 2FDG (2-[^18^F]-2-deoxy-D-glucose) and positron emission tomography (PET) to image [14]. PET is a noninvasive imaging method that permits high temporal and spatial resolution of ^18^F-labeled tracers in animal and human subjects. 2FDG enters tumors through GLUTs, where it is accumulated after conversion to 2FDG-6-phosphate by intracellular hexokinase. Although it has been envisaged that GLUT1 inhibitors could be used to block tumor growth, this has not advanced owing to the fact that GLUT1 is essential in the supply of glucose to the brain. Another challenge is that there are 14 genes in the human GLUT gene family SLC2, and specific isoform specific inhibitors have not yet come to market [13].

The cancer field has been slow to recognize that there is a second class of human glucose transporters, the sodium-coupled transporters, SGLTs in the SLC5 gene family [11, 22, 24]. It is well known that SGLTs are important in intestinal glucose absorption and glucose reabsorption from the glomerular filtrate in the renal proximal tubule [see 7, 18, 19, 24]. In addition to the small intestine and kidney, SGLT1 is expressed in the liver, lungs, brain, and salivary glands, while SGLT2 is limited to the renal proximal tubule [6, 11, 12, 17, 21, 24]. Since 2FDG is not a substrate for SGLTs, this means that 2FDG PET overlooks the potential contribution of SGLTs to glucose uptake into cells. We have developed a specific PET tracer for SGLTs, α-methyl-4[^18^F]-4-deoxy-D-glucopyranose (Me4FDG), and established that SGLT2 is functionally expressed in some tumors [10, 20, 22].

## SGLT2

**Fig. 1:**
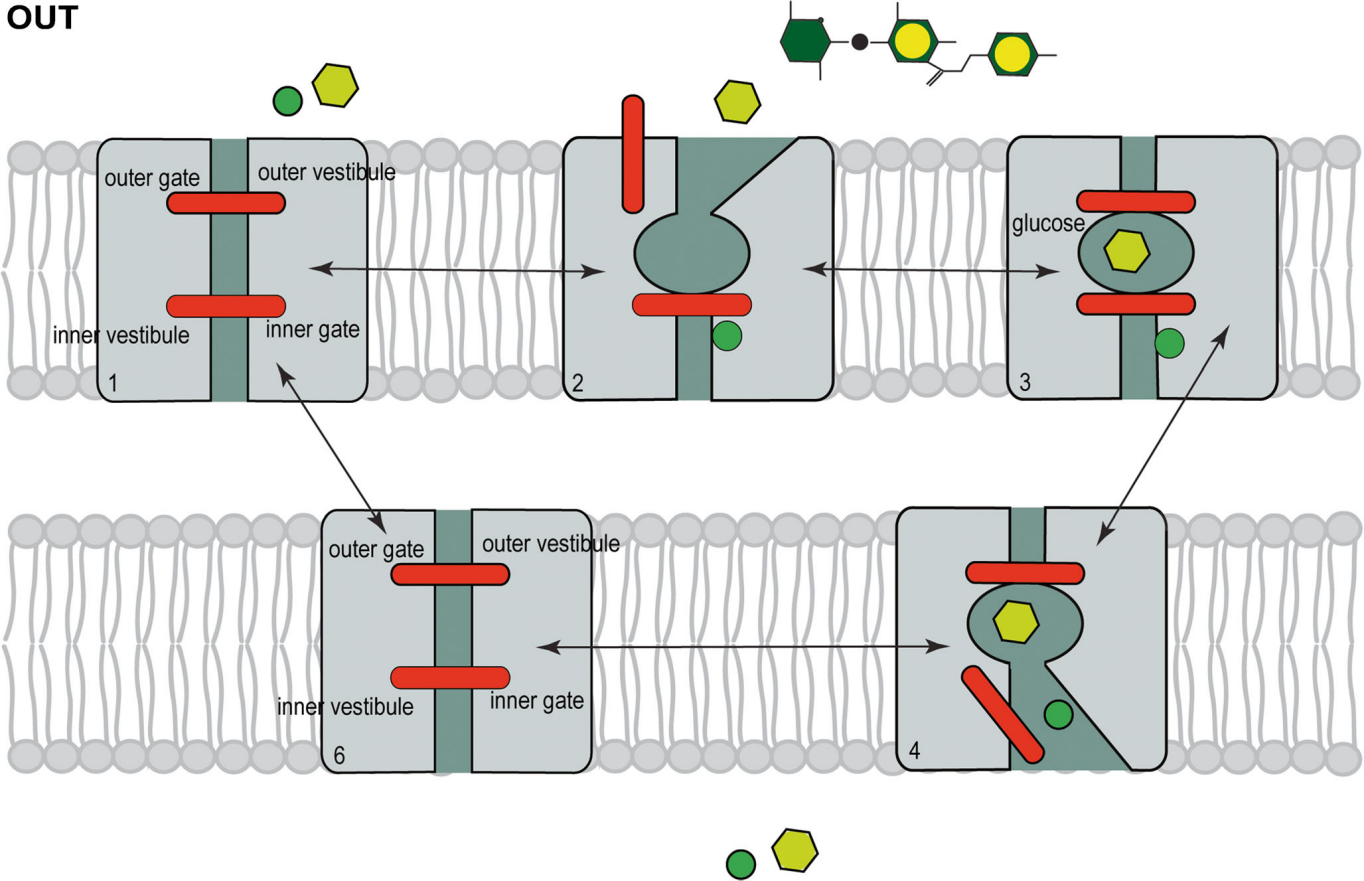
Model for SGLT2 sodium glucose cotransport. The integral membrane protein is shown with external and internal gates that control the entry and exit of glucose to the sugar binding site. External Na^+^ (solid green circle) first binds to open the outer gate to permit external glucose (yellow hexagon) to bind. The external gate then closes to occlude glucose from the external surfaces of the membrane, before the inner gate opens to allow Na^+^ and glucose to exit into the cytoplasm. The unloaded transporter finally returns to the starting outward conformation. There is obligatory coupling of one Na^+^ ion and one glucose during the transport cycle. The transporter is reversible with a turnover number of 50/s, but the rate and direction of transport depend on the Na^+^ and glucose concentration on each side of the membrane and the membrane potential. The natural glucoside phlorizin (green and yellow hexagons) blocks transport from the external side by binding to the glucose binding site in the presence of sodium [see 2, 5, 6, 9]

SGLT2 transports glucose in the kidney by Na^+^ cotransport with a Km of 5 mM (Fig. 1). The natural glucoside phlorizin is a non-transported competitive inhibitor that acts from the extracellular side of the membrane to block cotransport with a K_i_ of 11 nano-molar [2, 5, 8]. Phlorizin was the lead compound that the pharmaceutical industry used to develop specific SGLT2 inhibitors, e.g., dapagliflozin (Forxiga®) and empagliflozin (Jardiance®), to treat type 2 diabetes mellitus [3, 15].

## SGLT activity

The substrate specificity of SGLTs is distinct from that of GLUTs, e.g., α-methyl-D-glucopyranoside (αMDG) is transported by SGLT1 and SGLT2, but not GLUTs [23, 24]. For SGLT2 the K_m_’s for glucose and αMDG at 37 °C are 2, 5, and 6 mM [8]. The K_m_ for 2FDG is in excess of 300 mM [19]. This led Bruce Hirayama and I, in collaboration with Jorge Barrio and Vladimir Kepe, to develop Me4FDG as a specific ^18^F tracer to monitor SGLT activity in animal and human subjects [25]. Since the equatorial –OH at C#2 on the pyranose ring is essential for transport by SGLTs, we switched ^18^F from C#2 to C#4 and alkylated the C#1 –OH group to restrict transport to the SGLTs. Unlike 2FDG, Me4FDG is not a substrate for hexokinase [26], so Me4FDG is only accumulated in cells by the activity of SGLTs driven by sodium electrochemical potential gradient across the cell membrane. How do we distinguish between SGLT 2 and SGLT1 activity? This is accomplished by measuring the sensitivity of SGLT activity to specific, high-affinity SGLT2 drugs such as dapagliflozin, which are more than 300-fold more potent against SGLT2 than SGLT1 [3, 9]. The validity of dapagliflozin-sensitive Me4FDG uptakes by hSGLT2 has been confirmed using heterologous expression systems such as HEK293T cells [9].

Specific SGLT1 and SGLT2 polyclonal antibodies have been developed, tested, and verified in mice and human [1, 12, 20, 21, 24, 26]. The test for antibody specificity in immunohistochemistry includes absorption by their antigenic peptides and the use of SGLT1-/- and SGLT2-/- null mice. These antibodies were then employed to examine SGLT location in tumors in mice and humans [10, 20]. It should be noted that commercial SGLT antibodies have been problematic in immunocytochemical studies (12, 24). The distribution of SGLT2 in mice was confirmed using 4-[^18^F18]fluoro-dapagliflozin autoradiography and micro-PET [6], but the PET approach was not successful in humans due to rapid metabolism of the tracer.

## SGLT expression in tumors

The incentive to examine SGLT expression in tumors came about with our early use of Me4FDG PET in human subjects. These experiments confirmed the specificity of the tracer in that Me4FDG did not enter the brain and that it was not excreted into the urine [24]. Control experiments on the same subjects confirmed that 2FDG enters the brain and is excreted into the urine, due to the expression of GLUT1 in the blood-brain barrier and the fact that it is not a substrate for renal SGLTs. We included a few cancer patients in these early studies and found that Me4FDG was accumulated in glioblastomas and in metastatic prostate cancer.

The next chapter opened when SGLT2 antibodies and inhibitors became available, and we decided to study SGLT expression in fresh tumor samples collected from the operating room using protocols developed by Amy Yu and Bruce Hirayama [20, 22, 27]: First, we used viable tissue sections. Second, we measured SGLT activity using Me4FDG uptake and autoradiography to localize accumulated tracer in the heterologous tumor samples. Third, the activity was measured in the presence and absence of SGLT inhibitors. Fourth, the location of SGLT activity in the tumor was determined by SGLT immunohistochemistry.

**Fig. 2:**
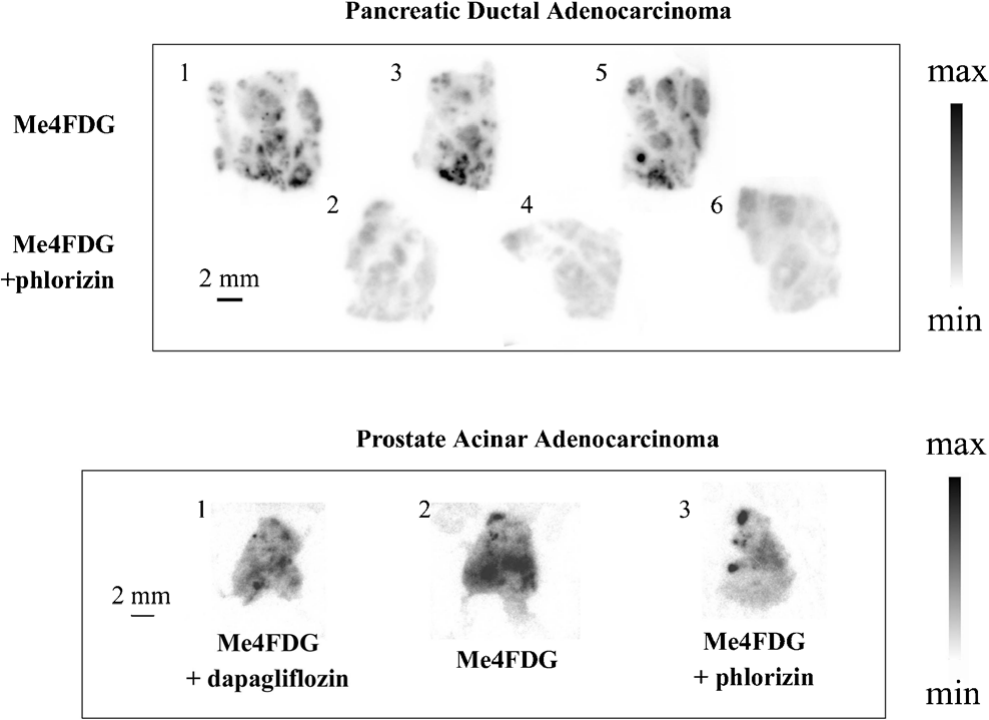
SGLT-dependent Me4FDG uptake in slices of human pancreatic and prostate adenocarcinomas. Fresh surgical cancer specimens were sectioned into 300-μm-thick slices and incubated for 20 min at 37 °C in oxygenated physiological buffer with 100 uCi of Me4FDG with and without 100 uM phlorizin or 250 nM dapagliflozin. Immediately afterwards the slices were washed with Me4FDG free cold buffer and exposed to autoradiographic plates. The numbers represent the order in which the slices were cut and assayed. Taken from Scafoglio et al. [20]

Examples of Me4FDG uptakes into serial sections of fresh pancreatic and prostate tumors incubated in the presence and absence of SGLT inhibitors are shown in Fig. 2. In each case, there were hot spots of Me4FDG uptake where the intensity was reduced by the inhibitors.

**Fig. 3:**
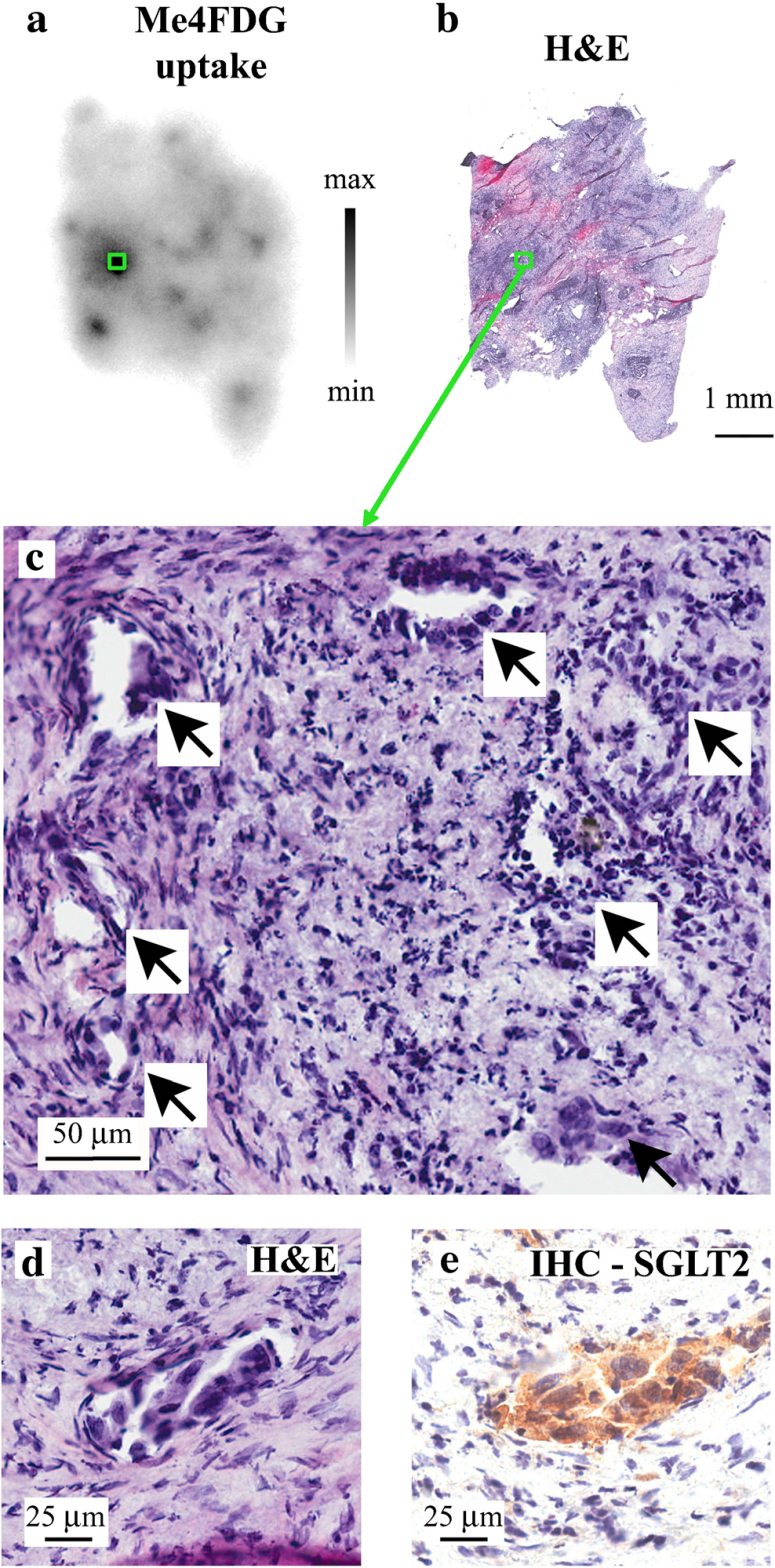
Correlation between Me4FDG uptake, morphology, and SGLT2 expression in a pancreatic adenocarcinoma. This experiment was conducted on a representative sample of a moderately differentiated pancreatic adenocarcinoma freshly harvested from a patient, and it shows predominant spots of Me4FDG tracer uptake. An autoradiographic image of Me4FDG uptake into the tumor sample is shown in Fig. 2a. The image shows one prominent hot spot representing high Me4FDG uptake. After the decay of radioactivity (^18^F half-time 109 min), the tissue was re-sliced into 10 μm thin sections and used for histology (hematoxylin and eosin) and SGLT2 immunohistochemistry. (**b**) A thin section of the tumor shown in **a** stained with H&E. The green square highlights the hot spot in **a**. (c) A higher magnification image of the hot spot, green square in **b**. This shows malignant ducts (arrows) in a background of fibrotic tissue and chronic inflammatory cells. (d) A representative malignant duct with pleomorphic cells, a high nucleus/cytoplasm ratio, and angulated appearance. (**e**) SGLT2 IHC on the malignant duct in **d** with strong immunoreactivity positivity in the malignant cells. This immunoreactivity was blocked by the SGLT2 antigenic peptide (not shown). Taken from Scafoglio et al. [20]

The anatomical location of hot spots revealed by autoradiography was determined by cutting 10 μm fixed sections of tumors after Me4DG radioactivity decayed (half-time 109 min) and staining sections with hematoxylin and eosin (Fig. 3b, c, and d) or with SGLT antibodies (Fig. 3e). The H and E sections of a tumor hot spot in this sample of pancreatic tumor, green square in Fig. 3b, show typical malignant ducts, arrows Fig. 3c, within a background of fibrotic and inflammatory tissue. At higher magnification, the malignant ducts are composed of angular pleomorphic cells with heavy eosin staining of their stratified nuclei (Fig. 3d). Finally, the same malignant duct was stained with SGLT2 antibody (Fig. 3e) showing immunoreactivity that was blocked by the antigenic SGLT2 peptide (not shown). SGLT1 immunohistochemistry gave light nuclear staining in the malignant ducts and no reaction in the desmoplastic regions. Similar results were obtained in nine pancreatic ductal adenocarcinomas. In the case of prostate adenocarcinomas, similar hot spots of Me4FDG activity were seen on autoradiographs (Fig. 2), and this was correlated with SGLT2 staining in the characteristic malignant microacini with a single layer of irregular epithelial cells [20]. Again staining was predominantly cytoplasmic, whereas light SGLT1 staining was nuclear. The resolution of light microscopy did not permit the exact subcellular distribution of SGLTs in our tumor sample, but the accumulation of Me4FDG sensitive to dapagliflozin points to at least some functional SGLT2 protein in the plasma membrane. While it is very difficult to estimate the transport activity of SGLTs detected by immunocytochemistry, we postulate that SGLT2 in malignant ducts may be responsible for tracer Me4FDG accumulation in tumor slices. The functional importance of SGLT2 expression in tumor cells is difficult to evaluate without knowing the maximal velocity (*V*_max_) of SGLT2 and GLUT1 of glucose uptake into malignant ducts.

## In vivo mouse models

As a segue to Me4FDG PET imaging in patients, we expressed pancreatic and prostate cell lines, ASPC-1 and PC-3 cells, in an NSG xenograft mouse model [20]. The tumors were evaluated using micro-PET, ex vivo autoradiography, and immunohistochemistry. In mouse micro-PET Me4FDG was distributed throughout the body except for the brain and urinary bladder. The uptake was not SGLT mediated in the heart, skeletal muscle, or liver, but SGLT2 and SGLT1 accounted for reabsorption from the glomerular filtrate [6, 16, 18]. In the mouse model, Me4FDG accumulated in the vital regions of the pancreatic and prostate tumors, and an oral dose of 1 mg/kg dapagliflozin reduced this by 40–50%, an amount comparable with the effect on urinary excretion. Ex vivo autoradiography showed that Me4FDG accumulated in the vital region of both tumors but not in the necrotic core. SGLT immunohistochemistry revealed specific SGLT2 staining in the vital regions of the tumors, but no SGLT1 staining. The expression of GLUTs was not examined by either 2FDG micro-PET or immunohistochemistry as it is well-nigh impossible to meaningfully compare Me4FDG and 2FDG micro-PET or SGLT and GLUT immunohistochemistry: 2FDG is accumulated by conversion to 2-FDG-6-phosphate and, as currently practiced, immunohistochemistry is not quantitative in terms of GLUT and SGLT functional activity.

Preliminary trials were then conducted to test the effect of SGLT2 inhibitors on pancreatic tumor growth and necrosis. Mice were orally treated with 30 mg/kg of either canagliflozin or dapagliflozin for 3 weeks, and tumor growth was measured between weeks 2 and 3. Canagliflozin reduced tumor growth significantly by 30%, but dapagliflozin did not. However, both drugs significantly increased necrosis by 70–100%. The difference between the two inhibitors on tumor growth is difficult to rationalize, especially given that the oral dapagliflozin was 200-fold higher than the prescribed dose for type 2 diabetes mellitus patients, 0.14 mg/kg. These results were tantalizing, but the differences between the two SGT2 drugs are difficult to explain.

## Me4FDG PET in cancer patients

**Fig. 4:**
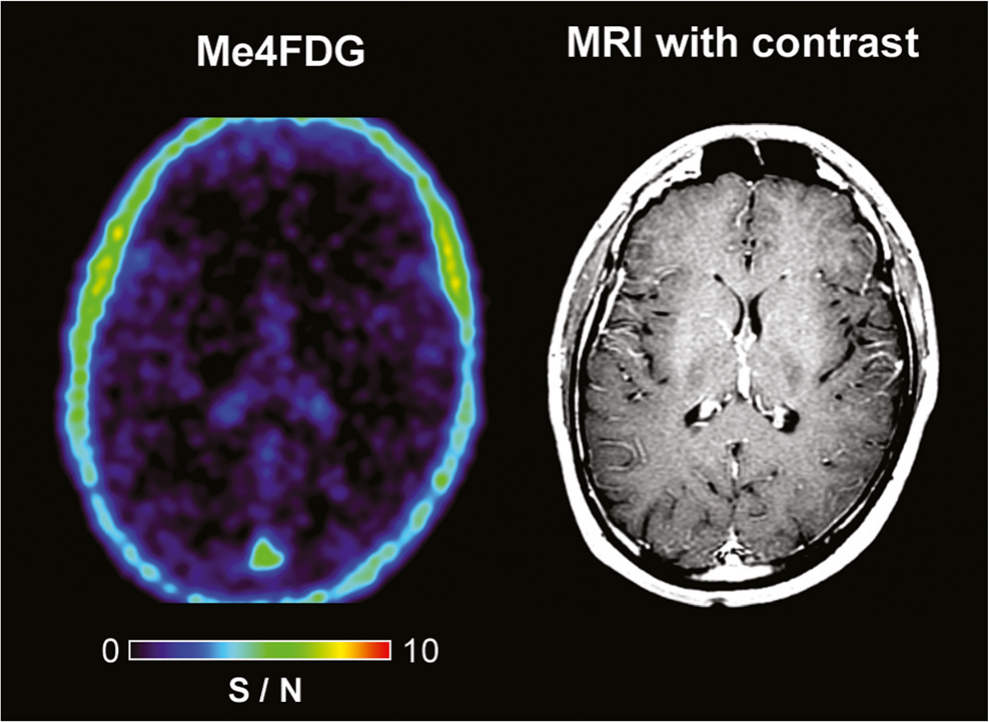
Me-4FDG and MRI scans on a control subject. Scans were conducted on a 24-year-old male patient with a history of epilepsy. The MRI (T1-weighted MP-RAGE with and without gadolinium contrast) was carried out as part of his clinical care. The brain Me4FDG PET scan 30 min after intravenous injection of 370 MBq Me4DG was conducted on a Siemens Biograph PET/CT scanner. The SUVR/BG scale, 0–10, is shown for the NIH color scale. Revised from Kepe et al. [10]

We have conducted Me4FDG PET scans on human subjects, including eight glioblastoma patients, three pancreatic cancer patients, and several prostate cancer patients. In healthy people, Me4FDG did not enter the brain as expected because of the absence of SGLTs in the normal blood-brain barrier and the fact that Me4FDG is not a substrate for GLUT1 in the blood barrier (Fig. 4) [24]. While Me4FDG does not enter the brain across the blood-brain barrier, it should be noted that at least in rodents, functional SGLTs are expressed throughout the brain, including the cortex, cerebellum, and hippocampus [26, 27]. Another major difference between Me4FDG and 2FDG PET is that the Me4FDG is not excreted in the urine (see [24]). There was minor Me4FDG accumulation in the skeletal muscle, kidney cortex, and liver, but, at least in mice, this was not due to SGLT1 or SGLT2 activity [18, 19].

**Fig. 5:**
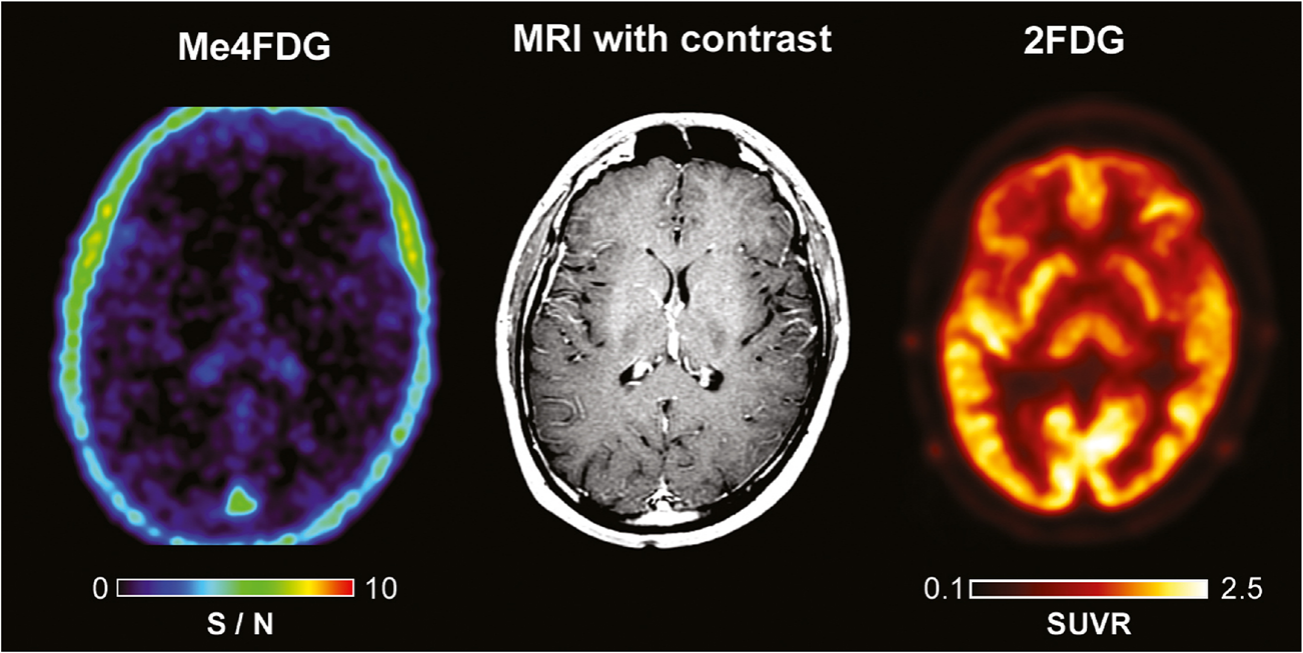
Me4FDG PET, MRI, and 2FDG PET scans on a WHO grade IV astrocytoma patient. The 57-year-old male patient has a 46-mm posterior corpus callosum astrocytoma. The Me-4FDG scan was conducted as for that on the control subject (Fig. 4) and shown as the S/N (SUVR/BG) ratio relative to the torcula on the NIH color scale. The standard clinical 2FDG PET brain scan is s for the “hot iron” on the SUVR based on a reference region of the brain. Taken from Kepe et al. [10]

In patients with WHO stage III and IV glioblastomas, Me4FDG does enter the brain across the blood-tumor barrier and accumulates in active tumor (Fig. 5). For comparison, the figure also shows the MRI and 2FDG PET images for this patient. The MRI images indicate that the blood-tumor barrier is disrupted for gadolinium, as is likely for Me4FDG. In dynamic PET scans, there was no evidence for Me4FDG washout from the tumor, indicating that indeed the tracer was accumulated in active tumor and not simply by passive entry into the tumor through the blood-tumor barrier. Me4FDG was restricted to the tumor, but 2FDG uptake was seen in the tumor and throughout the brain, especially in regions of high metabolic activity such as the cerebral cortex. Similar results were obtained on eight other patients with WHO stage III or IV glioblastomas. In general, the level of Me4FDG in tumors was 14 times higher than the background level in the brain parenchyma and 2 times higher than in blood. In terms of the resolution, tumors as low as 6 mm in diameters have been detected [10]. In the three patients with pancreatic adenocarcinomas studied so far, we were unable to detect any Me4FDG tumor accumulation.

**Fig. 6:**
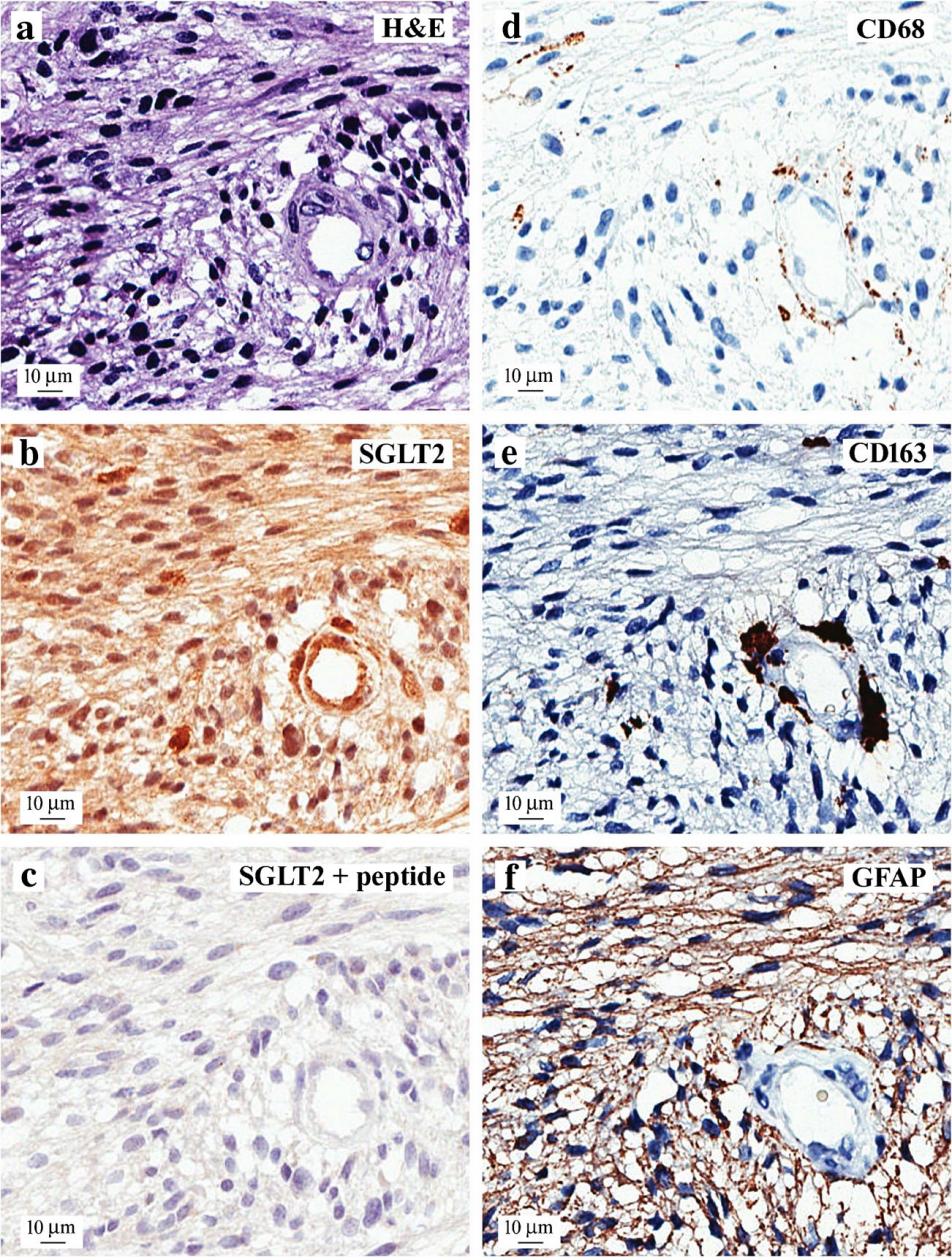
SGLT2 expression in a representative sample of a human glioblastoma. This frontal lobe WHO grade IV tumor was excised from the patient, and frozen sections were archived. Pathology reported a tumor with typical high cellularity, necrosis, and microvascular proliferation. Shown are adjacent sections of the tumor surrounding a blood vessel that were stained with hematoxylin and eosin (**a**) and antibodies for SGLT2 in the presence and absence of antigenic peptide (**b** and **c**), glial markers CD68 and CD163 (**d** and **e**), and an astrocyte marker GFAP (**f**). SGLT2 polyclonal antibody showing SGLT2 expression in a blood vessel endothelium, microglia/macrophages surrounding the blood vessel, and cancer cells that was blocked by the antigenic peptide. CD68 and CD163 staining was mostly restricted to microglia/macrophages surrounding the blood vessel, while diffuse GFAP immuno-positivity was in neoplastic cells. There was light SGLT1 staining of nuclei throughout the sample (not shown). Taken from Kepe et al. [10]

Which SGLT is responsible for Me4FDG uptake into glioblastomas? Our first approach towards answering this question was to examine SGLT1 and SGLT2 expression using immunohistochemistry. Figure 6 gives an example of the results we obtained on a WHO grade IV tumor excised from a patient that exhibited typical pathology of high cellularity, necrosis, and microvascular proliferation. Shown is the distribution of the immunoreactivity obtained for SGLT2, glia, and neoplastic cells in serial sections of a blood vessel surrounded by anaplastic cells (Fig. 6a). SGLT2 was found in the neoplastic cells throughout the tumor and in microglia/macrophages surrounding the vessel and in endothelial cells (Fig. 6b), and the SGLT2 antigenic peptide blocked this immunoreactivity (Fig. 6c). CD68 and CD163 staining was mostly restricted to perivascular surrounding the blood vessel (Fig. 6d and e), and diffuse GFAP staining (Fig. 6f) suggests the glial origin of the neoplastic cells. Light SGLT1 immunoreactivity was observed in nuclei throughout the tumor (not shown), but the significance of this is not known. At least in mouse models of pancreatic and prostate tumors, SGLT1 does not contribute to glucose uptake (20), and we plan to test this in patients using SGLT inhibitors.

**Fig. 7:**
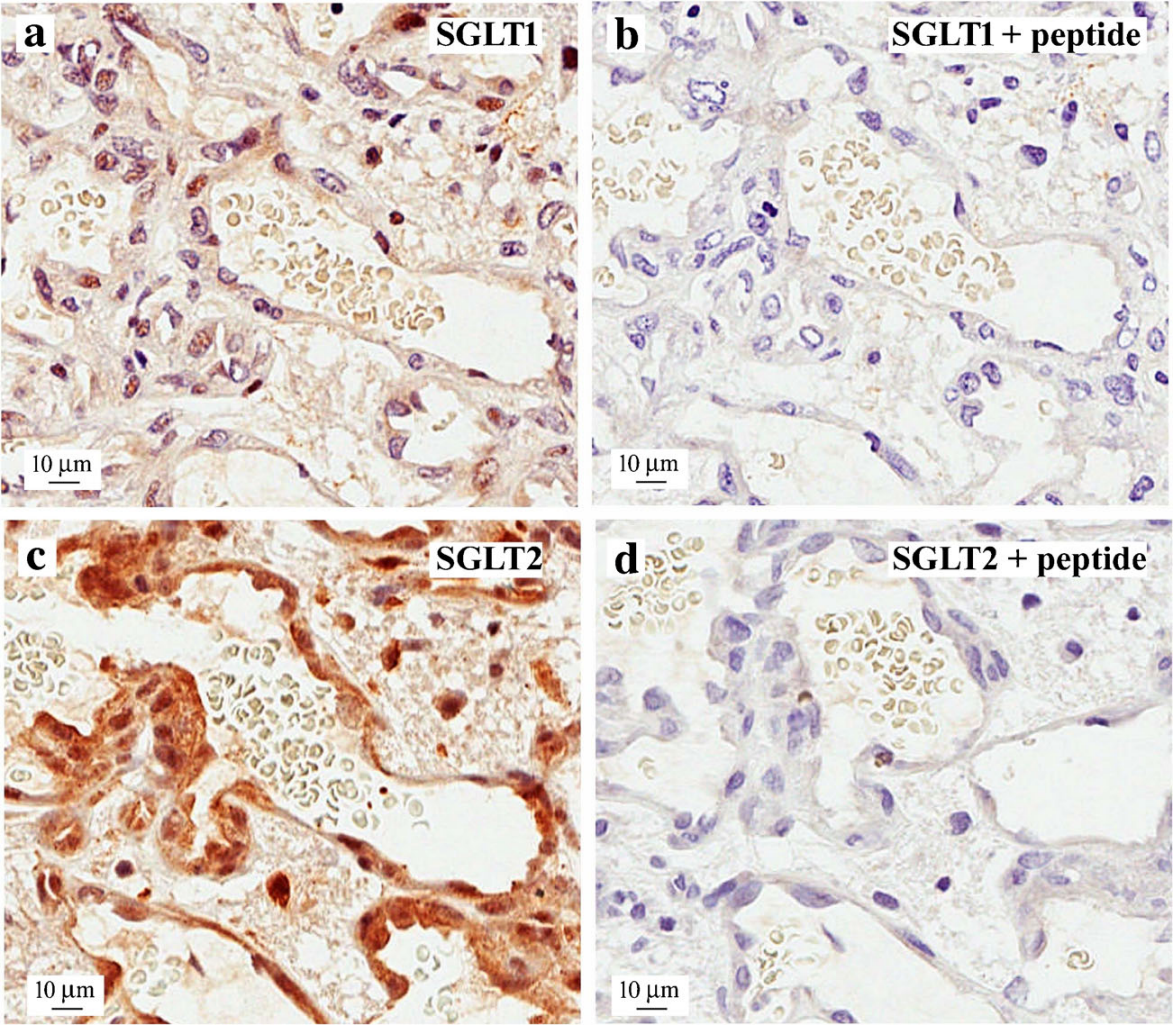
Expression of SGLT2 in a microvascular proliferation in a glioblastoma. A representative sample of human WHO grade IV glioblastoma showing the characteristic, irregularly shaped microvascular proliferation with (**a**) SGLT2 immunostaining and (**b**) SGLT2 immunoreactivity in the presence of the SGLT2 antigenic peptide. Note the intense specific staining of the endothelial cells with the SGLT2 antibody and the presence of unstained red blood cells in the lumen of the microvessel. There was light nuclear SGLT1 staining throughout the sample (not shown). Taken from Kepe et al. [10]

Another view of SGLT2 immunohistochemical location in the microvascular region of this tumor is shown in Fig. 7. There was robust staining of the thin endothelium of the microvasculature and surrounding tumor (Fig. 7a), and this blocked the SGLT2 immunogenic peptide (Fig. 7b). In some tissue samples, we were able to detect SGLT2 immunoreactivity at or near plasma membranes, and this suggests that endothelial SGLT2 participates in Me4FDG uptake across the blood-tumor barrier. SGLT2 is not expressed in the endothelium of the normal blood-brain barrier as judged by the lack SGLT2 immunoreactivity and Me4FDG uptake [10]. There was only a light specific labeling of endothelial nuclei by our SGLT1 antibody, but the significance of nuclear SGLT staining is not known at this time.

## Critical appraisal of Me4FDG accumulation in tumors

Our studies clearly show that tracer Me4FDG is accumulated in pancreatic and prostate tumors in vitro and high-grade glioblastomas in vivo, and this is correlated with the immunochemical detection of SGLT2. At least in our mouse models of pancreatic and prostate tumors, SGLT2 inhibitors reduce Me4FDG uptakes at normal plasma glucose levels indicating significant glucose uptake by SGLT2.

In the case of glioblastomas, the blood-tumor barrier is compromised, as seen for gadolinium uptakes into tumors in MRI scans (compare Figs. 4 and 5). This is not a major route of Me4FDG entry into brain tumors as there was no washout of Me4FDG from the tumors in dynamic scans lasting up to 2 h. So Me4FDG uptake into tumors is not simply due to breakdown of the blood-tumor barrier. SGLT2 is expressed in endothelial cells of the blood-tumor barrier, but not the normal blood-brain barrier, indicating that SGLT2 may play a part in Me4FDG transport across this barrier. I have no information about expression of SGLT2 in blood-tumor barriers in other tumors such as pancreatic adenocarcinomas.

We estimate that tracer Me4FDG is accumulated in tumors some 14-fold higher than normal brain parenchyma and up to 2.5-fold higher than in blood, probably due to Na^+^/Me4FDG cotransport through SGLT2. How can the significance of this be evaluated in patients? One possibility is to test the effect of SGLT2 inhibitors as we have done in mice. These drugs are fast acting and very specific, with a high affinity for SGLT2, K_1_ 1–5 nano-molar. In our hands, a single oral 25 mg dose of Jardiance inhibits the renal reabsorption of Me4FDG in human subjects within minutes, as does oral gavage and intravenous injection of dapagliflozin in mice [20. 6]. Given that oral Jardiance may block Me4FDG uptake into glioblastomas, what is the significance of such a finding? This is determined by the relative importance of GLUTs and SGLT2 in feeding glucose into tumors to support glycolysis. Biochemically, the answer to this question requires knowledge of the kinetics of glucose uptake by each transporter in tumor cells, i.e., the *K*_m_ and *V*_max_ for GLUTs and SGLT2. This is difficult to evaluate, but perhaps a definitive answer will come clinical trials to test the effect of SGLT2 drugs on tumor growth. Such trials are feasible given the success over the past 5 years in using SGLT2 drugs to inhibit renal glucose reabsorption in patients with type 2 diabetes mellitus. Clinical trials could be optimized by first confirming that tumors are positive using Me4FDG PET scans. Based on the studies presented here, candidate patients for treatment are those with WHO stage III and IV glioblastomas. Preliminary findings in patients also suggest that breast and metastatic prostate tumors may also respond to SGLT2 drug therapy.

Finally, there is an apparent discordance between our functional data and that in TCGA databases for SGLT expression in tumors. One explanation may be that functional activity and mRNA levels have not been measured in the same tumors, and another may be that SGLT mRNA levels do not provide direct information about functional expression. In one model system, the sheep small intestine, we have reported that diet can change SGLT1 activity by up to 200-fold, with only minimal changes in mRNA levels [see 24]. Again, clinical trials will resolve this issue.

## Summary

SGLT2 Na^+^/glucose cotransporters are expressed in human pancreatic and prostate adenocarcinomas as judged by in vitro Me4FDG uptake assays and immunocytochemistry and by mouse in vivo models as shown by Me4FDG micro-PET, ex-vivo autoradiography, and immunocytochemistry. In patients, Me4FDG uptake into high-grade glioblastomas was also observed in PET studies, and this was correlated with positive SGLT2 immunocytochemistry on malignant cells and tumor vasculature. At this time, the significance of SGLT2 expression in tumors is not known, but at a minimum, Me4FDG PET offers significant advantages over conventional 2FDG PET in imaging advanced glioblastomas (see Fig. 5). Studies are underway to test the effect of SGLT2 drugs on glucose uptake into tumors in patients, and this may lead to clinical trials to test SGLT2 drug therapy for tumors.
